# The diagnostic value of DMSA scan in differentiating functional pseudo-tumors from malignancies in scarred kidneys: case series and literature review

**DOI:** 10.1186/s12882-023-03113-5

**Published:** 2023-05-26

**Authors:** Enas Hussein Mohammed, Ahmad Kaddourah, Noor Al Khori, Mehdi Djekidel

**Affiliations:** 1 Department of Pediatrics, Division of Nephrology and Hypertension, Sidra Medicine, Doha, Qatar; 2 Department of Pediatrics, Weill Cornel Medicine University, Doha, Qatar; 3 Department of Radiology, Division of Body Imaging, Sidra Medicine, Doha, Qatar; 4 Department of Radiology, Division of Nuclear Medicine, Northwell, New York, USA

**Keywords:** Renal regenerating nodule_1_, Renal pseudo-tumor_2_, Chronic kidney disease_3_, Dimercaptosuccinic acid scan, DMSA_4_, Single photon emission computed tomography, SPECT_5,_, case series_6_

## Abstract

**Background:**

The terms “renal regenerating nodule” and “nodular compensatory hypertrophy” are used in the literature to describe functioning pseudo-tumors (FPT) in the setting of an extensively scarred kidney. FPTs are usually discovered incidentally during routine renal imaging. Differentiating these FPTs from renal neoplasms is critical but can be challenging in the setting of chronic kidney disease (CKD) given the limitations related to using contrast-based imaging.

**Case summaries:**

We report a pediatric case series of 5 CKD patients, with history of urinary tract infections, in which tumor-like lesions evolved in scarred kidneys and were incidentally discovered on routine renal imaging. These were diagnosed as FPT by utilizing dimercaptosuccinic acid (DMSA) imaging and showed stable size and appearance upon follow-up with ultrasound and MRI.

**Conclusion:**

FPTs can be picked up on routine imaging of pediatric patients with CKD. Although larger cohort studies are needed to confirm these conclusions, our case series supports the evidence that DMSA scan showing uptake at the site of the mass can be a useful tool to suggest the diagnosis of FPTs in children with kidney scarring, and that SPECT DMSA scan adds more precision in picking up and accurately localizing FPTs compared to planar DMSA.

## Background

Urinary tract infections (UTIs) in children can be severe enough to cause renal scarring and chronic kidney disease (CKD). Classically, the mature kidney is considered to have limited cellular regenerative capacity [1, 2]. However, this concept has been challenged by many studies in which biological evidence was introduced suggesting the ability of the kidneys to endogenously regenerate [1, 3–6]. There are a few reports describing the development of parenchymal functioning tumor-like masses in scarred kidneys. [7–10]. Although some reports describe these functioning pseudo-tumors (FPT) as “regenerating nodules”, their nature and whether they are newly regenerated renal tissue or an entity of focal compensatory parenchymal hypertrophy [7, 8] is a matter of scientific debate.

In the literature, the terms “renal regeneration nodule” and “nodular compensatory hypertrophy” are used to describe functioning or histologically normal renal masses that develop in the setting of scarred kidneys. For simplicity and to avoid confusing terminology, we elect to call these functioning tumor-like lesions “FPT” throughout the manuscript.

The radiologic characterization of these FPT poses a clinical dilemma and a diagnostic challenge. Conducting further clinical investigations to rule out malignancies in these lesions might subject patients to radiation exposure, complications of tissue sampling and possible surgical interventions in addition to the imposed psycho-economic factors such as anxiety and high costs [11]. Moreover, the underlying CKD necessitates avoiding contrast studies which limits the radiologic options needed to evaluate these masses [12]. In patients with CKD, ultrasound (US), non-contrast computed tomography (CT), and conventional magnetic resonance imaging (MRI) are frequently inconclusive in differentiating malignancies from FPT [13]. On the other hand, Dimercaptosuccinic acid (DMSA) scan uses a non-nephrotoxic radiotracer that is picked up by functioning renal nephrons, thus offers a plausible methodology to diagnose FPTs which have a relatively normal to increased radiopharmaceutical uptake, while neoplasms will display an area of decreased or absent uptake [7, 10, 14, 15]. Single photon emission computed tomography (SPECT) provides 3D reconstructions of the imaged kidney, leading to definite localization of the suspected mass and especially useful when multiple small FPT’s are present in a complex scarred kidney architecture. SPECT images can be fused with images obtained from the patient’s corresponding CT or MRI study and hence add specificity [16, 17]. Compared to planar DMSA, SPECT DMSA increases the sensitivity in regard to picking up more abnormalities that might be less detectable on 2D planar images that represent multiple planes superimposed on each other and displayed as a 2D image. [17, 18].

In this work, we report our experience with five pediatric CKD cases in whom tumor-like lesions were radiologically identified in severely scarred distorted kidneys. The main objective of this work is to highlight the role of DMSA scans with single planar and SPECT images in suggesting the diagnosis FPT and ruling out malignancy. This should facilitate the clinician’s management of these patients. We additionally review the available literature to discuss the possible pathophysiology of developing these FPT in the scarred kidneys.

## Case presentations

This retrospective case series included all children with established CKD whom incidental finding of tumor-like lesions were discovered upon routine renal imaging. All patients were recruited from Sidra Medicine, the only tertiary children’s hospital in the state of Qatar.

### Case 1

**Fig. 1:**
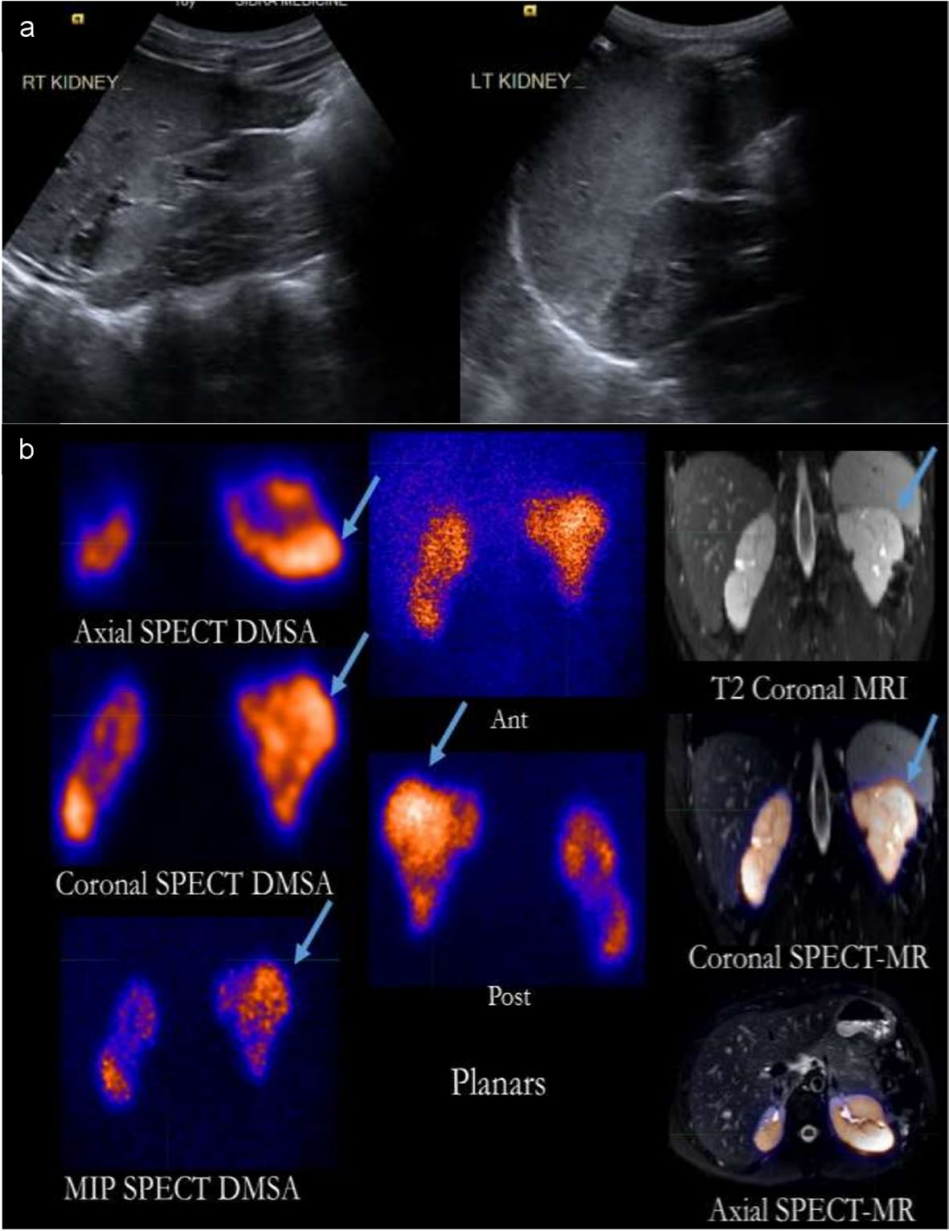
**A** US images of both kidneys demonstrate bilateral irregularity of the renal contours and multiple areas of renal scarring. **B** 16-year-old boy with CKD and bilateral abnormal kidneys on US. A large nodule is shown with a blue arrow on SPECT DMSA images and correlated on MRI of the abdomen. Increased background uptake is also noted on DMSA scans because of poor underlying renal function. Uptake in this nodular mass in the upper pole of the left kidney is diagnsotic of a FPT. Abbreviations: MR: Magnetic resenonace. MIP: Maximum intensity projection

A sixteen-year-old boy presented with fever, loin pain and dysuria. He had a history of recurrent febrile illnesses without associated urinary symptoms. Physical Examination (PE) showed a normotensive thriving young man and no palpable abdominal masses. Urine analysis was negative for leucocyte esterase, nitrites and white blood cells at the time of presentation. Work up revealed creatinine of 122 mcmol/L with estimated glomerular filtration rate (eGFR) of 51 ml/min/1.73m2. Renal US showed bilateral hyper-echogenic kidneys with evidence of scarring (Fig. 1**(A)**). MRI of the abdomen and pelvis was done and showed diffusely abnormal renal parenchyma, multiple scars, irregular outline with undulations and adjacent protrusions of the parenchyma resembling a mass like structure on the left kidney (Fig. 1 (B)). SPECT DMSA scan showed a large nodular mass representing FPT (Fig. 1 (B)). Voiding cystourethrogram showed right grade 3 vesico-ureteric reflux (VUR) with normal bladder and urethra. A clinical diagnosis of frequent missed or subclinical urinary tract infections (UTIs) was proposed. Bilateral ureteral deflux implant procedure was done. He had a stable e-GFR during follow-up and no evidence of urinary tract infections (UTIs). 2 years later, follow-up Renal US did not show any interval changes in the appearance of both kidneys.

### Case 2

**Fig. 2:**
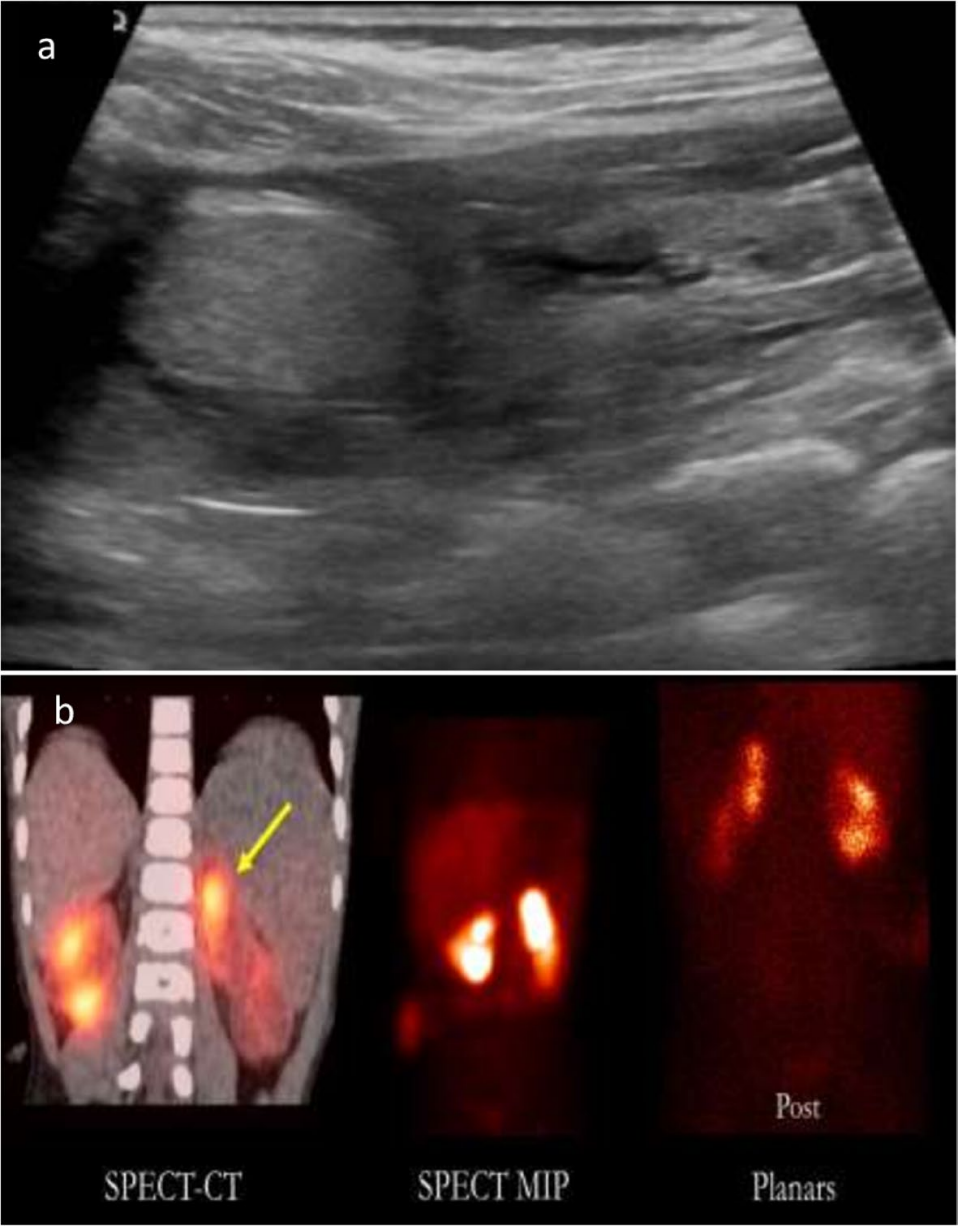
**A** US image of the left kidney shows an echogenic renal mass in the left upper pole. **B** 2-year-old boy with dysplastic kidneys and an echogenic left renal pseudo-tumor on US. The mass is best seen on 3D SPECT-CT and SPECT-MIP images compared to planar images showing radiotracer uptake and found to be a FPT. Split renal function was also decreased on the left to 37%

A two-year-old boy with a known history of posterior urethral valve had a valve ablation and bilateral ureterostomies in the neonatal period with subsequent development of CKD stage 4 and a history of recurrent UTIs. A routine US (Fig. 2 (A)) that was done for the evaluation of the pelvi-calyceal system prior to ureterostomy closure showed an incidental finding of a left renal mass that was not reported on previous US. Parents denied history of increased irritability, change in urine smell or color and particularly no hematuria. Physical exam showed an afebrile, thriving, normotensive boy and no abdominal masses. To evaluate the mass, a two-dimensional (2D) planar DMSA scan was done as well as three-dimensional (3D) SPECT imaging of the kidneys (Fig. 2 (B)). The mass was diagnosed as a FPT, and its size was static on subsequent follow up US studies 18 months later. No further interventions were required.

### Case 3

**Fig. 3:**
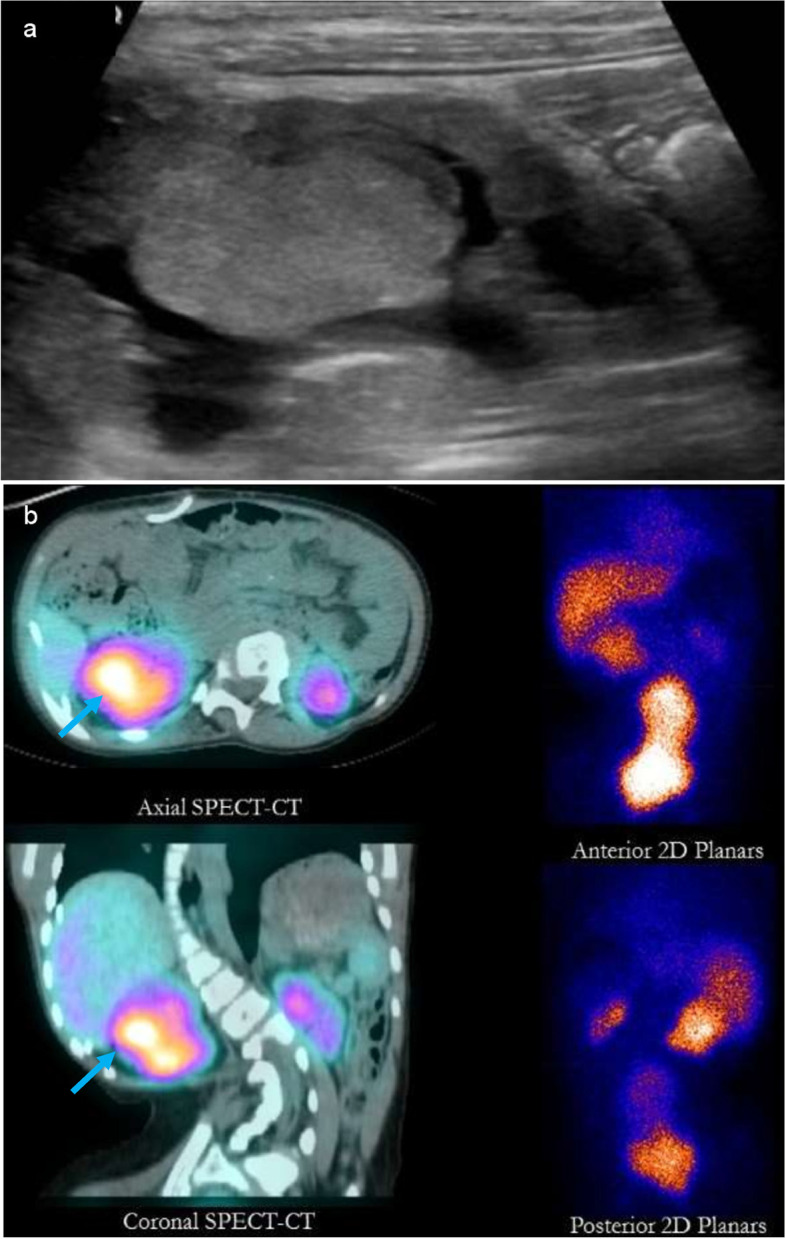
**A** US images show a large echogenic renal mass on the right. Both kidneys showed loss of cortico-medullary differentiation and cortical defects in keeping with renal scars. **B** 9-year-old girl with neurogenic bladder and severe bilateral renal scarring and renal masses on US (the largest of which is showed in Fig. 3 (A)). Those masses are showing uptake of DMSA in a pattern consistent with FPT on the right side. Uptake is barely seen on 2D planar imaging. In this case, 3D SPECT-CT (white arrows) offers better localization and improved sensitivity, specificity and reader confidence compared to 2D planar imaging. This is even more evident in this case with severe spinal deformity

An eight-year-old girl with spina bifida and neurogenic small-capacity urinary bladder was managed with bladder augmentation, Metrofenoff creation and clean intermittent catheterization (CIC). Her course was complicated with frequent UTIs and CKD stage 3. An US done to work up urine leakage around the Metrofenoff site showed an incidental finding of multiple areas of parenchymal irregularities in the right kidney suspicious of renal masses (Fig. 3 (A)). She had no abdominal pain, abdominal swelling, urinary symptoms or hematuria. Physical exam showed a normotensive afebrile girl who was failing to thrive and had no abdominal masses. DMSA showed FPT pattern more evident on SPECT compared to planar images (Fig. 3 (B)). The masses were stable in size and appearance compared to an US done 10 months later.

### Case 4

**Fig. 4:**
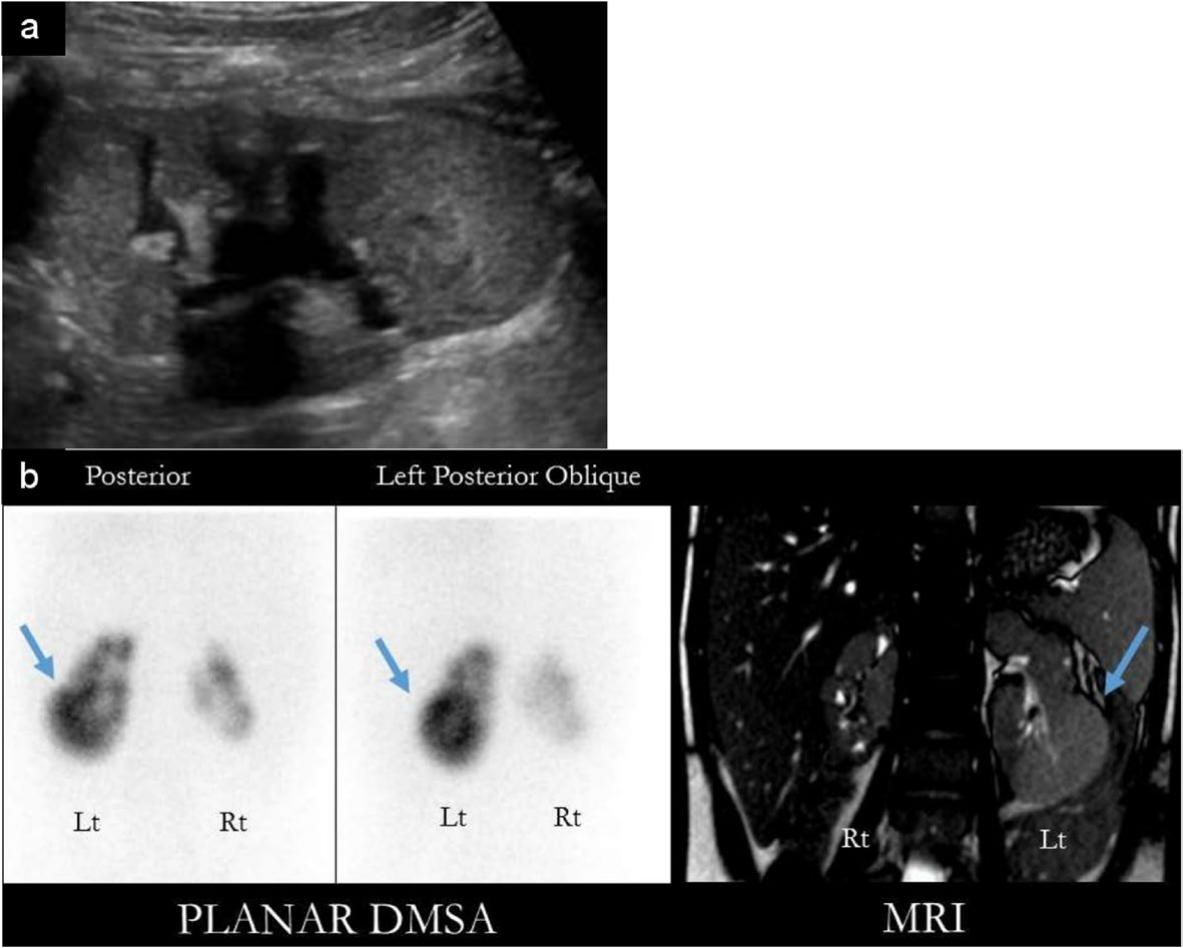
**A** US images showing a mass-like lesion in the lower pole of the left kidney. **B** DMSA study showing a FPT in the left lower pole corresponding to the site on MRI (blue arrow)

A nine-year-old girl presented with poor weight gain for a 2-year-duration. Her history was significant for 2 episodes of UTI which were investigated at the time clinically with no imaging. Physical examination revealed elevated blood pressure and failure to thrive. Work up showed creatinine of 79 mcmol/L consistent with eGFR of 60 mL/min/1.73 m^2^ and proteinuria. Urine analysis was negative for leucocyte esterase and nitrites. Renal US showed a left lower pole hyperechoic mass-like lesion (Fig. 4 (A)). She was afebrile with no urinary symptoms. She didn’t have hematuria, abdominal distension, or a palpable abdominal mass. She was admitted to the hospital for work up of the mass. MRI of the abdomen and pelvis confirmed the presence of the left renal mass (Fig. 4 (B)). A DMSA scan was done to evaluate whether this mass contains normal functioning renal tissue and confirmed radiotracer uptake at the site of the mass suggesting the diagnosis of FPT. Split renal function was 79% on the left and 21% on the right (Fig. 4 (B)). VCUG showed a right grade 3 VUR and left grade 1 VUR. She was diagnosed with CKD stage 3 due to reflux nephropathy and stage 2 hypertension. 1 year later, a repeat MRI showed stable size and appearance of the left renal mass.

### Case 5

**Fig. 5:**
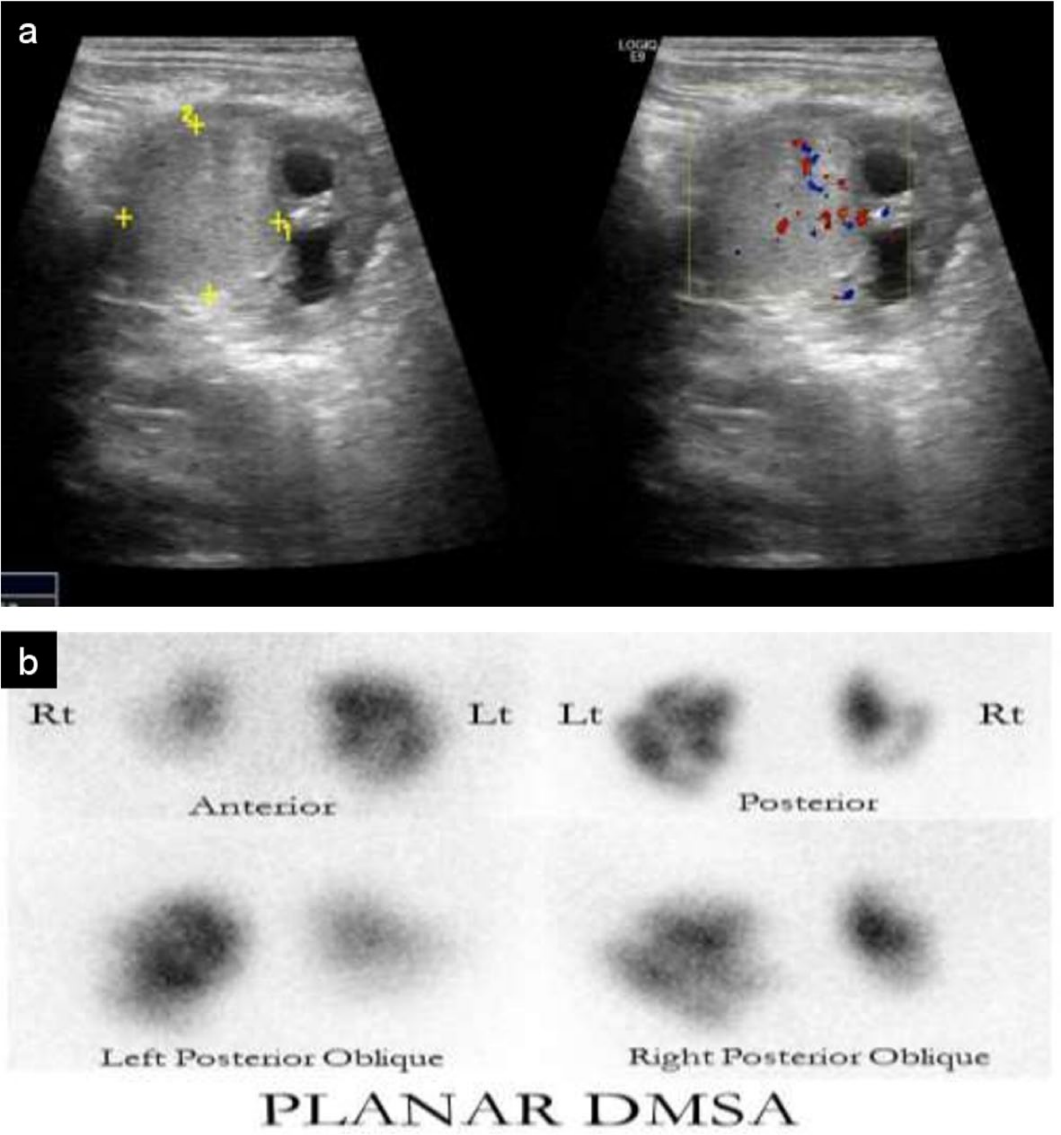
**A** US image shows a right renal mass. On color Doppler, the mass demonstrates internal vascularity. **B** DMSA scan images show multiple areas of scarring in both kidneys as well as nodular uptake in the right kidney most consistent with FPT (arrow)

Our last case is a 5-year-old girl with sacral agenesis, neurogenic urinary bladder, bilateral grade 3 VUR and CKD stage 3. She had recurrent episodes of UTI despite being on clean intermittent catheterization and UTI antimicrobial prophylaxis. Vesicostomy was planned. Pre-operative renal US was done to evaluate the degree of hydronephrosis and showed a new finding of a right upper pole renal mass that was occupying most of the kidney parenchyma (Fig. 5 (A)). This mass was not reported/seen on the US scan done a few months prior. In the period between the 2 US scans, she had 2 symptomatic UTIs. She was admitted to the hospital for renal mass work up. No urinary symptoms suggestive of UTI, hematuria, abdominal pain or palpable masses were reported. DMSA scan showed focal tracer uptake in the mid-pole of the right kidney and a tracer defect in the upper pole (Fig. 5 (B)). The nodule on the ultrasound was presumed to correspond to the focal uptake on DMSA scan suggesting the diagnosis of FPT. However, SPECT DMSA; which would have allowed better characterization of the mid-pole uptake and the right upper pole defect; was not done. After vesicostomy, she had no further episodes of UTI. The mass was followed up with serial renal US studies that didn’t detect an increase in size over a two-year duration. However, a new mid-pole hyperechoic nodule was detected in the left kidney on a follow up US and showed tracer uptake on repeat DMSA scan.

## Results

In this work, we present five cases of pediatric CKD patients (Table 1) in whom incidental tumor-like masses were discovered on their scarred kidneys. Those masses were classified as FPTs after showing radiotracer uptake on DMSA scans. All patients had episodes of UTI and four of them had VUR.

**Table 1:** Patient characteristics at the time of the incidental renal mass detection

Patient	Age in years	Gender	eGFR in ml/1.73 m^2^/minute	History of UTI	VUR	Out-come
1	16	Male	51	Y	Y	Stable mass size on US 2 years after the mass was first detected
2	2	Male	26	Y	Y	Stable mass size on US 18 months after the mass was first detected
3	8	Female	44	Y	N	Stable mass size on US 10 months after the mass was first detected
4	9	Female	62	Y	Y	Stable mass size on MRI 1 year after the mass was first detected
5	5	Female	39	Y	Y	Stable mass size on US 2 years after the mass was first detected

Three of the reported cases had established CKD and were following up regularly in the nephrology clinics. The FPT were not seen early-on in the course of their disease and were only noted on later US scans.

In all cases, FPT were incidentally discovered, hence, work-up was initiated to rule out a renal neoplasm. None of the patients had hematuria, abdominal distension or palpable abdominal masses that might clinically suggest malignancy. 2 patients had poor weight gain. All the five cases were subjected to additional work-up in the form of blood tests and further imaging. In all cases, DMSA imaging especially when paired with SPECT played a substantial role in identifying those lesions as functional pseudo-tumors. All patients were followed-up either with US scans or MRI and showed stable appearance and size of the FPT (Table 1). However, one patient has developed a new mass on the other kidney which in turn showed tracer uptake on DMSA scan. 3 of our patients had SPECT-DMSA imaging which allowed more precision in picking up the FPTs and in their localization compared to planar DMSA as shown in Fig. 1 (B), 2 (B) and 3 (B).

## Discussion and conclusion

Determining the underlying pathology of newly discovered nodules or masses in patients with established CKD represents a diagnostic challenge. Malignancy, despite being relatively rare, is among the top differential diagnosis of those masses. Working-up FPT might expose the patients to prolonged medical, radiologic, interventional workup and psycho-economic burdens before revealing their underlying benign pathology [11, 12, 19]. In this work, we present five pediatric CKD patients who had incidental findings of tumor-like lesions that were suggested to be functional renal tissue by utilizing DMSA imaging with better precision when coupled with SPECT imaging compared to planar DMSA. Those FPTs were later on followed up by US and were stable in size and appearance (Table 1).

All five cases had a history of recurrent episodes of UTI and renal scarring. Four of them had VUR. These findings are in agreement with other case reports with similar presentations of UTI and VUR [9, 20].

Three of the reported cases had established CKD and were followed up regularly in the nephrology clinic. The FPT were not seen early-on in the course of their disease and were only noted on later US scans. Whether FPTs represent newly formed renal tissue (i.e., regenerating nodules) or a rare entity of focal compensatory hypertrophy and whether those two mentioned mechanisms represent a spectrum of renal regeneration is a question that needs further cellular-based investigations.

The mechanism by which the kidney restores acute or chronic lost function has been a debatable topic. Different mechanisms were suggested such as;

The presence of circulating extra-renal stem cells that can differentiate and integrate into the existing renal tissue to restore function [3]. These cells have the ability to go through numerous cycles of cell division while maintaining an un-differentiated state (i.e. self-renewal ability) and to generate a progeny of differentiated cells or their precursors (i.e. multipotent characteristic) [21]

The presence of multipotent intra-renal progenitor cells [1, 4–6]. These cells have the ability to differentiate along one or more particular cell lineages, but display a limited self-renewal potential [21].

Mature resident renal cells undergoing de-differentiation, migration into the areas of damage, and re-differentiation to replace the neighboring dead cells [1, 4].

Compensatory kidney hypertrophy (CKH) is a well-described physiologic phenomenon, through which the kidney cells increase in size (hypertrophy) but not in count (hyperplasia) [22]. It contributes to kidney growth and restoration of kidney function in the setting of reduced total nephron mass, such as in a single functioning kidney, or in the remaining kidney tissue following unilateral radical or partial nephrectomy [22–27]. While the mechanisms underlying CKH have been well-reported in the literature [22], such compensatory phenomena are debatable and not well-reported in significantly scarred kidneys. Two main mechanisms have been proposed to explain CKH. First, after kidney injury, the remaining functioning renal nephrons increase their activity (hyper-filtration) and undergo hypertrophy. Second, release of a renal specific factor that initiates CRH in response to loss of functioning kidney tissue [22].

Few papers have reported the findings on kidney biopsies of FPT. In one case series, the ultrasound-guided renal biopsies of the FPT showed normal glomeruli and tubules that are double or triple the usual diameter, without fibrosis [7]. Another case report documented a mass lesion that histologically showed a segmental or regional compensatory hypertrophy [8].

All tumor-like lesions were discovered incidentally in our case series with subsequent additional work-up to identify the nature of the lesions. With the advancement and wide availability of different imaging modalities, the number of incidentally discovered kidney masses has significantly increased [28, 29]. Although this has led to early detection of kidney tumors [30], a significant percentage of these masses were benign in nature, leading to unnecessary stress, tests and even unnecessary biopsies and surgeries [11, 12, 19]. Interestingly, studies looking at patients who underwent resection of solitary kidney lesions, 11–30% of the masses showed benign pathology depending on the studied population [19].

The incidence of childhood renal and suprarenal neoplasms depends on the age of presentation. Neuroblastoma may be seen in the perinatal period. Benign renal masses predominate in early infancy. Wilms' tumor is the most common renal malignancy from infancy to adolescence. Renal cell carcinoma becomes more frequent towards adolescence [31]. Less commonly encountered renal neoplasms are medullary carcinoma, angiomyolipoma, and metanephric tumors. Lymphomas are usually multifocal masses, but has also been reported as a solitary mass [32].

A kidney pseudotumor is defined as a mass that mimics the appearance of a neoplasm radiologically but contains normal renal tissue histologically and requires no treatment [10, 15, 33].

Differential diagnosis of renal pseudo-tumors can be classified into:

Congenital causes including hypertrophied column of Bertin, persistent fetal lobulation, spleno-renal fusion and dromedary humps.

Acquired causes in cases of CKD, known in the literature as “regenerating nodules” or “focal nodules of compensatory hypertrophy” which we elect to call FPT.

Some authors include Wegener granulomatosis, renal pelvic hematomas, arteriovenous malformation and infections (such as xantho-granulomatous pyelonephritis, abscess, focal pyelonephritis, fungal and TB pyelonephritis) as a part of the differential diagnosis [15, 33], however those represent unique pathologic entities with secondary renal involvement [10].

The FPTs were not observed on prior US scans of the 3 patients with established CKD who were previously following up in our hospital, which makes congenital causes unlikely. None of the 5 patients had symptoms suggestive of UTI. Urine cultures were negative for bacterial and fungal micro-organisms making the diagnosis of focal pyelonephritis unlikely. The masses all showed significant tracer uptake on DMSA scan which suggests functioning renal tissue and makes other pathological conditions with secondary renal involvement such as hematomas, arteriovenous malformations, granulomas, sarcoidosis, and most importantly neoplasms less likely. All patients were followed-up with kidney US scans or MRI which showed stable appearance and size of the FPT which is not the expected course in malignancy (Table 1).

FPTs represent a diagnostic challenge and necessitate further radiologic work-up. Even after conducting US and non-contrast CT, some lesions still remain indeterminate [14]. In one study on CKD patients, 92.5% of the tumor-like lesions evaluated by conventional MRI remained indeterminate [13]. Contrast-enhanced CT and MRI are frequently unfavorable due to advanced CKD and the potential risk of contrast-induced-nephropathy and nephrogenic systemic fibrosis respectively. Hence, contrast-free and isotope-mediated imaging is plausibly preferable [13].

DMSA scan is non-invasive, non-nephrotoxic, readily available technique that usually does not require sedation [34]. It is specifically a renal cortical imaging modality used in the diagnosis of renal parenchymal disorders, mostly scarring. However, in this case series, we used DMSA scans to pick up normally functioning renal tissue (i.e., the FPTs) in the setting of severely scarred malfunctioning kidneys. The mechanism by which the radiotracer targets the renal cortex is not well established. The most widely accepted theory suggests that the radiopharmaceutical is bound to plasma proteins and freely filtered by the glomeruli and reabsorbed by renal proximal tubular cells accumulating in the kidney cortex. This indicates the need for both functioning glomeruli and cortical tubules for the isotope to be up taken in cortical tubules [14, 35, 36]. This can explain why there is decreased radiotracer uptake in patients with proximal tubular dysfunction despite normal gross anatomy and normal creatinine clearance [35, 37]. Accordingly, we argue that radiotracer uptake will be decreased or absent in any neoplasm even if it was highly differentiated since it lacks function.

Normally, the functioning renal tissue will pick up the radiotracer, while nonfunctioning tissues, such as renal scars, cysts or neoplasms will have decreased or absent uptake, thus appearing photo-penic [10, 14]. DMSA scans of our CKD patients revealed areas of relatively normal or increased uptake corresponding to the FPTs. Historically, DMSA was used more extensively to evaluate renal masses in the late 1970’s and 1980’s [38–41], however this practice has shifted to ultrasound, CT and MRI characterization. In previous case reports where biopsies of the FPT were done revealing functioning renal tissue, all nodules displayed radiotracer uptake on the scans done preceding excision [7, 8]. More recently Gruning et al. eluded to this concept in a series of 15 patients with renal masses and showed a high accuracy (100%) at excluding malignancy when uptake is present [14].

Three of our patients had undergone SPECT-DMSA imaging which allowed more precision in picking up the FPTs as shown in image 3 (B) and more accuracy in their localization compared to planar DMSA scans, as shown in Fig. 1 (B), 2 (B) and 3 (B). Planar 2D scans lack the ability of demonstrating 3D structures, even if images are taken in different planes [16]. SPECT provides 3D reconstructions, leading to definite localization of the suspected mass which helps in corelating it to the suspected mass seen on the other imaging modalities. Moreover, SPECT images obtained from gamma cameras can be fused with the patient’s corresponding CT or MRI study [16, 17]. In addition, SPECT DMSA increases the sensitivity regarding picking up more abnormalities that might be less detectable on planar images [17, 18]. However, the main disadvantage of SPECT is the increased scanning time which may be an important factor in the pediatric population [34]. This limitation can somewhat be overcome with newer faster scanners.

Other modalities that were similarly proposed as a contrast-free problem-solving tool in cases of pseudo-tumors in scarred kidneys are MRI with diffusion weighted images and contrast-enhanced US. On MRI with diffusion-weighted images, kidney tumors may show restricted diffusion while FPT would not show restricted diffusion [13, 15]. MRI with diffusion weighted images has its own technical and logistical challenges including possible requirement for sedation and requires more robust data. Contrast-enhanced US uses non-nephrotoxic microbubble contrast agents that can help characterize FPTs. However, unlike DMSA, contrast-enhanced US is not widely available and clinical expertise is still limited [42, 43].

In conclusion, FPTs can be picked up on routine imaging of pediatric patients with CKD. Although larger cohort studies are needed to confirm these conclusions, our case series supports the evidence that DMSA scans showing uptake at the site of a renal mass can be a useful tool to suggest the diagnosis of FPTs, and that SPECT DMSA scan adds more precision in picking up and accurately localizing and characterizing FPT compared to planar DMSA.

## Data Availability

All data generated or analysed during this study are included in this published article.

## Abbreviations

CKD: Chronic Kidney Disease
eGFR: Estimated glomerular filtration rate
FPT: Functional pseudo-tumor
CKH: Compensatory kidney hypertrophy
DMSA: Dimercaptosuccinic acid
SPECT: Single photon emission computed tomography
VUR: Vesico-ureteric reflux
UTI: Urinary tract infection
CT: Computed tomography
MRI: Magnetic Resonance Imaging
US: Ultrasound
3D: Three-dimensional
2D: Two-dimensional
